# Nanopore-Templated Silver Nanoparticle Arrays Photopolymerized in Zero-Mode Waveguides

**DOI:** 10.3389/fchem.2019.00216

**Published:** 2019-04-10

**Authors:** Donghoon Han, Garrison M. Crouch, Zhongmou Chao, Susan K. Fullerton-Shirey, David B. Go, Paul W. Bohn

**Affiliations:** ^1^Department of Chemistry, The Catholic University of Korea, Bucheon, South Korea; ^2^Department of Chemical and Biomolecular Engineering, University of Notre Dame, Notre Dame, IN, United States; ^3^Department of Chemical and Petroleum Engineering, University of Pittsburgh, Pittsburgh, PA, United States; ^4^Department of Aerospace and Mechanical Engineering, University of Notre Dame, Notre Dame, IN, United States; ^5^Department of Chemistry and Biochemistry, University of Notre Dame, Notre Dame, IN, United States

**Keywords:** zero-mode waveguide, photopolymerization, solid-polymer electrolyte, recessed Ag ring electrode, nanopore array

## Abstract

*In situ* fabrication of nanostructures within a solid-polymer electrolyte confined to subwavelength-diameter nanoapertures is a promising approach for producing nanomaterials for nanophotonic and chemical sensing applications. The solid-polymer electrolyte can be patterned by lithographic photopolymerization of poly(ethylene glycol) diacrylate (PEGDA)-based silver cation (Ag^+^)-containing polyelectrolyte. Here, we present a new method for fabricating nanopore-templated Ag nanoparticle (AgNP) arrays by *in situ* photopolymerization using a zero-mode waveguide (ZMW) array to simultaneously template embedded AgNPs and control the spatial distribution of the optical field used for photopolymerization. The approach starts with an array of nanopores fabricated by sequential layer-by-layer deposition and focused ion beam milling. These structures have an optically transparent bottom, allowing access of the optical radiation to the attoliter-volume ZMW region to photopolymerize a PEGDA monomer solution containing AgNPs and Ag^+^. The electric field intensity distribution is calculated for various ZMW optical cladding layer thicknesses using finite-element simulations, closely following the light-blocking efficiency of the optical cladding layer. The fidelity of the polyelectrolyte nanopillar pattern was optimized with respect to experimental conditions, including the presence or absence of Ag^+^ and AgNPs and the concentrations of PEGDA and Ag^+^. The self-templated approach for photopatterning high-resolution photolabile polyelectrolyte nanostructures directly within a ZMW array could lead to a new class of metamaterials formed by embedding metal nanoparticles within a dielectric in a well-defined spatial array.

## Introduction

The increasing utilization of micro- and nanostructured devices in chemical sensing and nanophotonic applications has created a need for precise spatial, temporal, and geometric control over the formation of nanoscale systems. For example, solid-polymer electrolytes, a particularly interesting class of materials due to potential applications in batteries and actively configurable systems, have been utilized in memory devices based on resistive switching (Lin et al., 2014). Further, active control of conductive filament formation and dissolution in solid electrolytes may provide the foundation for a new class of metamaterials with reconfigurable optical properties, especially when combined with arrays of metal nanoparticles embedded in the dielectric host. Previously, we demonstrated actively reconfigurable constructs based on solid-polymer electrolyte-based nanoelectrochemical systems for the formation and dissolution of metal conductive filaments in polyethylene oxide (PEO)-based (Wu et al., 2011; Crouch et al., 2017) and poly(ethylene glycol) diacrylate (PEGDA)-based (Chao et al., 2018) electrolytes. In addition, polymer electrolytes formed by photopolymerization of polyethylene glycol (PEG)-based electrolyte have been utilized for various biomaterials and biomedical applications (Mellott et al., 2001; Burdick and Anseth, 2002; Aimetti et al., 2009; Huebsch et al., 2014; DeForest and Tirrell, 2015). Several methods have been introduced to fabricate miniaturized polymer electrolyte features, including molding (Terray et al., 2002; Fairbanks et al., 2009), printing (DeForest and Anseth, 2012), and lithography (Jang et al., 2007). Combined with patterning of polymer electrolyte composites, *in situ* polymer electrolyte photolithography can be used to determine the spatial composition pattern, e.g., molecular weight, degree of cross-linking, in a polymer electrolyte, in addition to physical shape (Bong et al., 2010; Wu et al., 2015). All of these techniques provide degrees of micro- and nanoscale control over polymer electrolyte's physical/chemical properties, and geometric resolution of micro- and nanostructures on multiple length scales.

Photopolymerization is an attractive technique as it provides unparalleled spatial and temporal control over polymer electrolyte spatial composition patterns and fabrication characteristics. Diacrylate-based polymers are particularly appealing in this context, since they exhibit exceptional transparency, color variation, robust mechanical properties, and elasticity (Mark, 2013). Acrylates can be chemically cross-linked to form polymer electrolytes for a variety of applications and are widely used in industrial chemical processes as adhesives, sealant composites, and protective coatings (Mark, 2013). In contrast to other monomers, acrylates are attractive due to their biocompatibility, semi-permeability, and chemical versatility, allowing modification with a range of mono- or multifunctional moieties (Burkoth and Anseth, 2000; Metters et al., 2000). Of specific importance to the current studies, acrylate-based polymers can controllably produce cross-linked networks *via* photopolymerization (Yu et al., 2001). To exploit these useful characteristics, polyethylene glycol (PEG)-based photo-crosslinkable polymers, based upon acrylate polymerization chemistries, have been developed (Sawhney et al., 1993; Nguyen and West, 2002).

Photoinitiated polymerization of acrylates is typically performed in the presence of a photo-initiator (PI) which generates free radicals upon exposure to UV light. Photopolymerizable acrylates are typified by poly(ethylene glycol) diacrylate (PEGDA), an ion-conducting polymer (Yang et al., 2006) frequently used in biological applications (Shu et al., 2004). It provides design flexibility, because the material can start at low-viscosity and be converted to a high-viscosity solid simply by exposing it to light. In the current work, this property could support the precise positioning of metal nanoparticles, by first placing them in a liquid-like environment, after which the metal nanoparticles are locked into place by simple UV exposure.

Placement of well-defined metallic nanostructures within a dielectric material allows the optical properties of the composite material to be tailored, potentially achieving responses not possible in a single-component material (Oldenburg et al., 1998; Prasad, 2004; Rill et al., 2008; Shukla et al., 2010a,b). This capability is most notably exploited in the rapidly advancing field of metamaterials—composite materials that exhibit unusual optical and electromagnetic properties such as a negative refractive index (Smith et al., 2004; Kuwata-Gonokami et al., 2005; Furlani and Baev, 2009b). The unique properties of metamaterials arise from the engineered electromagnetic/optical response of subwavelength structures, for example well-defined nanoporous metallic arrays, rather than the intrinsic properties of the constituent materials. These metamaterials hold promise for various applications such as far-field subwavelength imaging, nanoscale optical trapping, ultracompact waveguides, and optical power limiting (Baev et al., 2007; Furlani and Baev, 2009a). Most experimentally-realized metamaterials have been fabricated by “top-down” lithography techniques, usually either e-beam lithography (EBL) or focused-ion-beam lithography (FIBL) (Kuwata-Gonokami et al., 2005; Lee et al., 2008; Rill et al., 2008; Henzie et al., 2009). Although powerful, these serial, direct-write approaches are not amenable to large-area patterning. Another approach to metamaterials involves laser direct-writing in a polymeric structure followed by metal deposition onto the fabricated surface (Shukla et al., 2010b). Although this method is promising, full metal coverage is challenging. Thus, new fabrication methods are needed.

Here we present a new approach for the fabrication of PEGDA-based solid-polymer electrolyte nanopillars constructed from silver nanoparticles (AgNPs) templated within recessed Ag ring electrode arrays. Importantly, the Ag ring electrodes are bifunctional, serving as the optical cladding layer in zero-mode waveguides (ZMWs) during fabrication while retaining the capability to serve as working electrodes for electrochemical tuning of the array in future applications. As ZMWs, the metal layers control the spatial distribution of optical radiation used in photopolymerization and, consequently, the distribution of cross-linked PEGDA photopolymer. This approach can significantly simplify the fabrication of PEGDA solid-polymer electrolyte arrays and provide a viable route to the fabrication of three-dimensional metamaterials by photopolymerization. Metamaterials can be realized by embedding AgNPs in a well-defined dielectric-embedded nanopore array, where the optical properties can be tuned by adjusting the number of AgNPs, the inter-pore spacing, or both. Here, we explore the controlled fabrication of ZMW nanopore-templated photopolymerization of AgNP-containing PEGDA polymeric structures. This study indicates that: (1) metal ZMW arrays may be used to template the nanopillars for the polymerization process; (2) the ZMW can be tuned to control the spatial distribution of the confined optical field and, thus, of the photopolymerization process; and (3) these properties can be realized in the presence of nanopore-embedded AgNPs and Ag^+^ ions in the electrolyte.

## Materials and Methods

### Chemicals

Anhydrous acetonitrile (ACN), silver nitrate (AgNO_3_), sulfuric acid (95%), hydrogen peroxide (30%), fluorescein 5(6)-isothiocyanate (FITC), and 2-hydroxy-4′-(2-hydroxyethoxy)-2-methylpropiophenone photoinitiator (PI) were obtained from Sigma-Aldrich. Silver nanoparticles (AgNPs, 50 nm size, 5 kDa PEG capped) were purchased from nanoComposix. Poly(ethylene glycol) diacrylate (PEGDA) with number average molecular weight *M*_*N*_ = 700 was purchased from Sigma-Aldrich. Cleanroom-cleaned glass coverslips (Glass D, 75 x 25 mm, 1.0 mm thick) were obtained from Schott Nexterion. All reagents were used as received.

### Fabrication of ZMW Arrays

**Figure 1:**
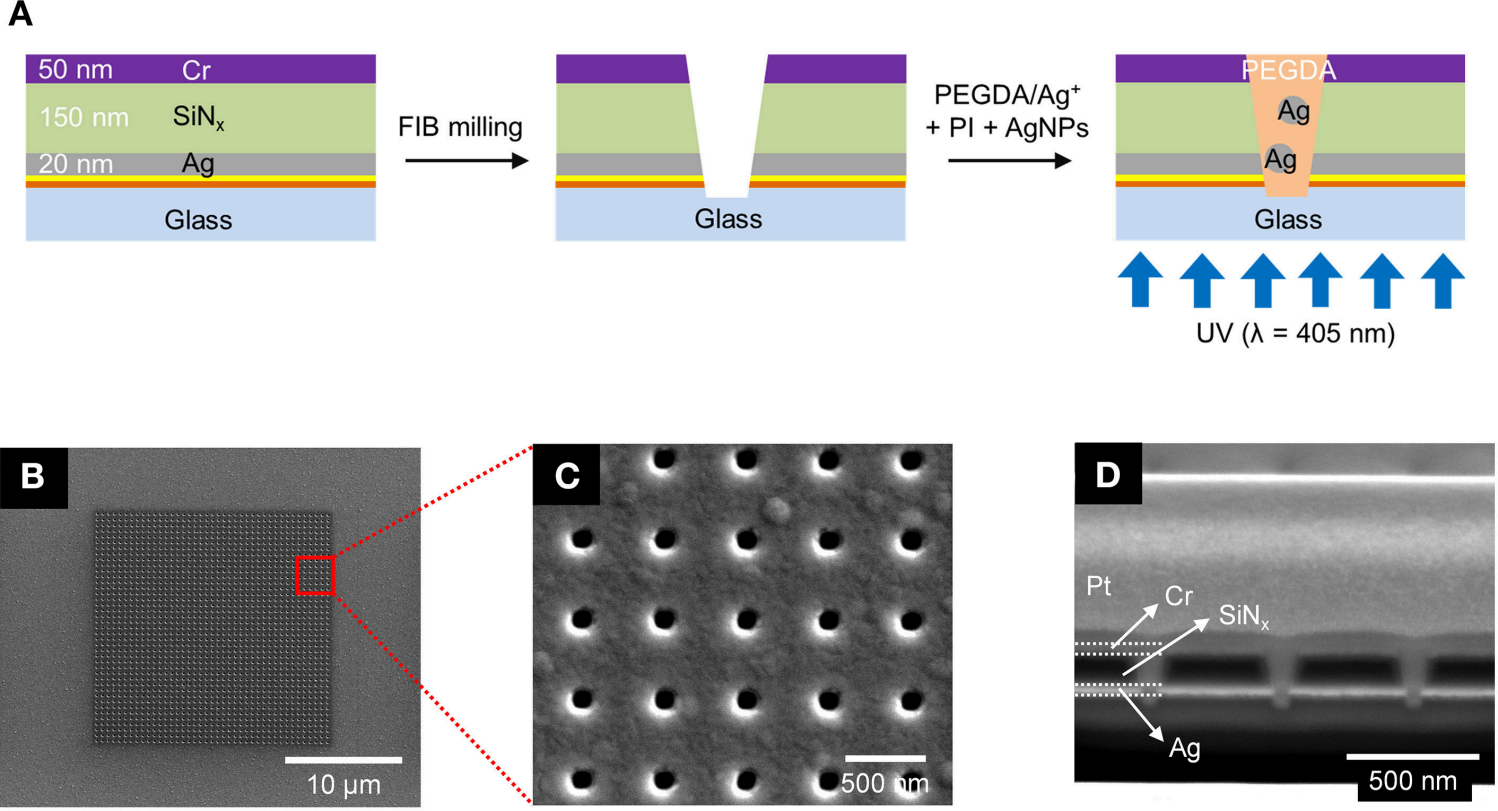
**(A)** Schematics of the fabrication process for the photopolymerized ZMW array. **(B–D)** Scanning electron micrographs of a nanopore array at different magnifications and perspectives. **(B)** Top-down view of the entire 20 × 20 μm nanopore array. **(C)** Top-down view of a 5 × 5 subset of the same array. **(D)** Cross sectional SEM image of the nanopores taken at 52° tilt.

The nanopore arrays were fabricated via a combination of standard photolithography, layer-by-layer deposition, and focused ion beam (FIB) milling. The primary processing steps are shown in Figure 1A. Glass slides were cleaned in piranha solution (3:1 sulfuric acid (95%):hydrogen peroxide (30%)—*Caution*—*Strong oxidizer, use with extreme care*), rinsed with deionized (DI) water, and dried at 120°C. A 5 nm thick Au layer was deposited by electron-beam evaporation (UNIVEX 450B, Oerlikon) after deposition of a 5 nm Ti adhesion layer. Then, a 20 nm Ag film was e-beam evaporated onto the same glass slide, after which a 150 nm thick SiN_x_ layer was deposited by plasma-enhanced chemical vapor deposition (PECVD 790, Plasma-Therm). Finally, an additional 50 nm thick Cr layer was deposited on the substrate. A dual-source FIB instrument (Helios Nanolab 600, FEI Corp.) was used for milling and characterization. Nanopore arrays were patterned in a 20 × 20 μm square array with a lattice spacing of 500 nm, shown in Figures 1B,C. FIB milling was performed at 30 kV acceleration, 0.28 nA ion aperture, and 0.1 ms dwell time to produce the recessed dual-ring electrode (RDRE) array. These FIB-milled pores exhibit a conical frustum shape with typical top diameter, *d*_*top*_~140–160 nm, and bottom diameter, *d*_*bottom*_~60–80 nm. Although milled under nominally identical conditions, small sample-to-sample variations in pore geometry were observed.

### Photopolymerization of PEGDA Solution

In an argon-filled glovebox with oxygen and water concentrations controlled to <0.1 ppm, 10 and 20 mM solutions of AgNO_3_ in anhydrous ACN were prepared. Similarly, PEGDA was dissolved in ACN to make 0.5, 1, 2, and 3 wt% PEGDA solutions, each adjusted to 1 mM of PI. To purify the AgNPs, 1,000 μL of 50 nm AgNPs was centrifuged at 10,000 rpm for 30 min (microcentrifuge RS-200, REVSCI); then the solvent was decanted, and the AgNPs were resuspended in 100 μL ACN. The AgNPs and AgNO_3_ solution were added to the PEGDA + PI solutions in a 1:1:8 volume ratio, yielding final solutions of AgNO_3_ in ACN with concentrations of 1 and 2 mM for PEGDA concentrations of 0.5, 1, 2, and 3 wt%, respectively. Fifty microliter of each solution was then dropcast onto the nanopore array inside the glovebox. The PEGDA-coated nanopore array was exposed to UV light (405 nm) with an intensity of 14 mW cm^−2^ for 30 min in the cleanroom. Next, the nanopore array was washed with ACN and dried in filtered air.

### Fluorescence Measurements

Fluorescence measurements were performed on an Olympus IX71 wide-field epi-illumination microscope. Radiation from a 488 nm laser was passed through an excitation filter (Chroma Z488/10X), and defocused to illuminate an area *ca*. 100 × 100 μm on the Cr side of the sample to directly excite fluorescence of FITC molecules in the nanopores. The fluorescence was collected by a 100 × NA 1.4 oil-immersion objective, and projected onto a 512 × 512 pixel CCD camera (Andor Technology Ltd). A dichroic mirror (Chroma Z488RDC) and emission filter (Chroma HQ525/50 m) were used to separate excitation from emission radiation.

### Modeling and Calculations

Finite element simulations were performed using COMSOL Multiphysics version 5.3. The simulations were performed over a two-dimensional domain representing the geometry and dimensions of the zero-mode waveguides employed in our experiments (Han et al., 2017). We used the “Electromagnetic Waves” physics of COMSOL in a frequency domain mode to obtain the excitation field inside a ZMW. A free triangular mesh was used with “Extremely fine” resolution and refinement applied to the ZMW layer. The ZMW was represented by a single pore, consisting of an adhesion layer (Ti, *h* = 5 nm), a second adhesion layer (Au, *h* = 5 nm), an optical cladding layer (Ag, *h* = 20, 50, and 100 nm), a dielectric layer (SiN_x_*, h* = 150 nm), and a top layer (Cr, *h* = 50 nm). A perfectly matched layer was incorporated in the glass substrate component to cancel any reflection artifacts from the simulation boundaries. The complex refractive indices of Ti (Werner et al., 2009), Au (Olmon et al., 2012), Ag (Werner et al., 2009), and Cr (Johnson and Christy, 1974) were taken as *n* = −5.74 + i4.4, −1.14 + i6.1, −4.70 + i2.5, and −4.13 + i11.8, respectively. The refractive indices of water, glass, and SiN_x_ were taken to be 1.33, 1.45, and 2.016, respectively. Excitation radiation at 405 nm wavelength, consistent with the UV aligner, was simulated to irradiate the bottom of the glass module, arriving perpendicular to the plane of the structure.

## Results and Discussion

### Characterization of ZMW Devices

ZMW arrays with 500 nm inter-pore spacing were fabricated to form square arrays consisting of annular apertures of sacrificial layer(Cr)-insulator(SiN_x_)-metal layer(Ag). In these recessed Ag ring electrode nanopore stacks, the Ag layer can be electrically connected and used as an electrode for electrochemical experiments. Figure 1A illustrates the fabrication process using standard photolithography, layer-by-layer deposition, and FIB milling to produce nanopore recessed Ag ring electrode arrays. This simple direct-write approach enables direct fabrication of precise nanopore structures exhibiting conical frustum shapes in contrast to the cylindrical nanopores obtained using electron beam (Dawson et al., 2012; Kleijn et al., 2012) or nanosphere lithography (Fu et al., 2018). Cylindrical pore shapes would likely result in minor alterations to the concentration parameters required for optimal pore filling due to changing in polymer wetting behavior, and the extent to which the photocrosslinking radiation can penetrate the pore. Figures 1B,C show SEM images of the entire array with an interpore distance 500 nm, and Figure 1D shows a corresponding cross-section SEM image of typical pores produced by FIB milling. The overetched region below the Ag/glass interface typically decreases with pore diameter, and well-controlled milling processes yield pores overetched by ≤50 nm. From bottom to top, the cross-section image in Figure 1D shows the bottom Ag layer (bright), silicon nitride (black), and the sacrificial Cr layer (bright). The nanopore ZMWs exhibit a conical shape with a larger aperture at the top than at the bottom electrode. The typical diameter of the top of the pore (*d*_top_) is ~140–160 nm, while the bottom diameter (*d*_bottom_) is ~60–80 nm.

### Effect of the Thickness of Ag Layer on UV Irradiation

The spatial distribution of the optical field, both within and external to the ZMWs, is important in determining the extent of photopolymerization that will occur within the nanopores. For wavelengths above the cutoff wavelength of the nanoaperture, λ_c_, where λ_c_ ~ 1.7 *d* and *d* is the pore diameter, the evanescent field decays exponentially with distance at a rate that depends on the radius and diameter (Crouch et al., 2018). In addition, the light-blocking efficiency of the Ag optical cladding layer also depends on the thickness of Ag. Experimentally, photopolymerization of PEGDA within nanopores was accomplished with collimated UV radiation from a UV aligner irradiating the bottom (Ag ring) surface of the ZMW array, as shown schematically in Figure 1A. To assess the effectiveness of this strategy in the ZMW nanopores studied here, finite element simulations were performed for various Ag thicknesses.

**Figure 2:**
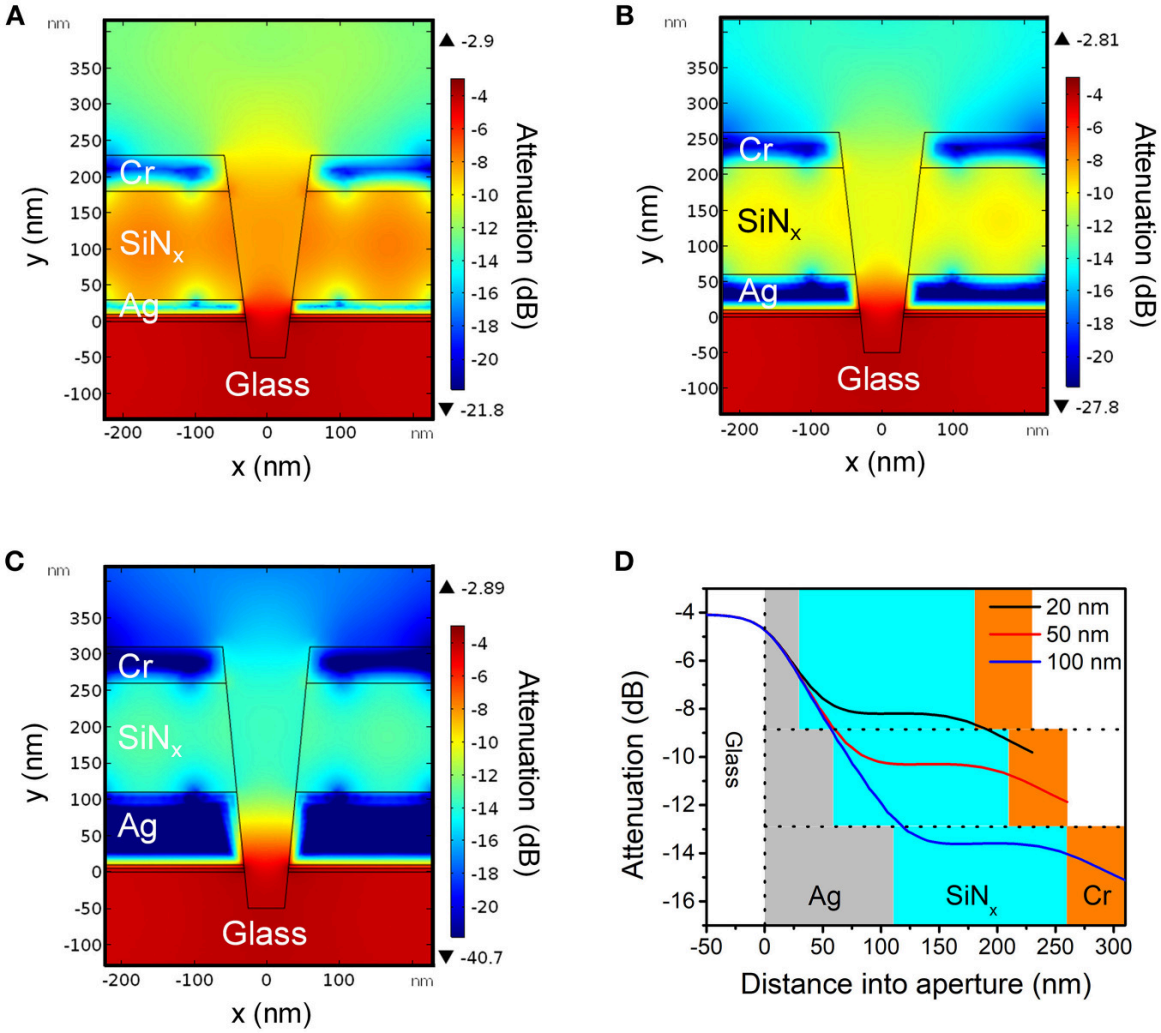
Cross-sectional heat maps of evanescent field amplitudes in conical nanopores obtained by finite element simulations for Ag layer thicknesses of **(A)** 20 nm, **(B)** 50 nm, and **(C)** 100 nm, respectively. For all cases, bottom aperture of the nanopore structure had a diameter of *d*_*bottom*_ = 70 nm and the top aperture was determined by the ratio *d*_*top*_/*d*_*bottom*_ = 2. **(D)** Simulated attenuation of energy density along the central axis of the ZMW for different thicknesses of Ag as indicated in the legend. Area shading corresponds to the material surrounding the opening: gray (Ag), blue (SiN_x_), orange (Cr).

Figures 2A–C show the electric field amplitudes in a series of conical frustum pores each having different Ag layer thicknesses (20, 50, and 100 nm, respectively), but with constant ratio *d*_top_/*d*_bottom_ = 2. As Figures 2A–C illustrate, the field decays exponentially within the nanopore, and the nanostructures provide attenuation primarily determined by the thickness of the Ag optical cladding layer, with thinner Ag thicknesses leading to less attenuation. Figure 2D shows the attenuation of electromagnetic energy density inside apertures with different Ag layer thicknesses, where for the 20 nm Ag thickness (black line), the smallest energy attenuation is predicted, confirming the thickness-dependent attenuation. These results indicate that thinner Ag thicknesses provide less optical field confinement and, thus, should lead to more spatially-extended PEGDA photopolymerization within the ZMW nanopores.

### Characterization of Photopolymerized PEGDA in ZMW Devices

**Figure 3:**
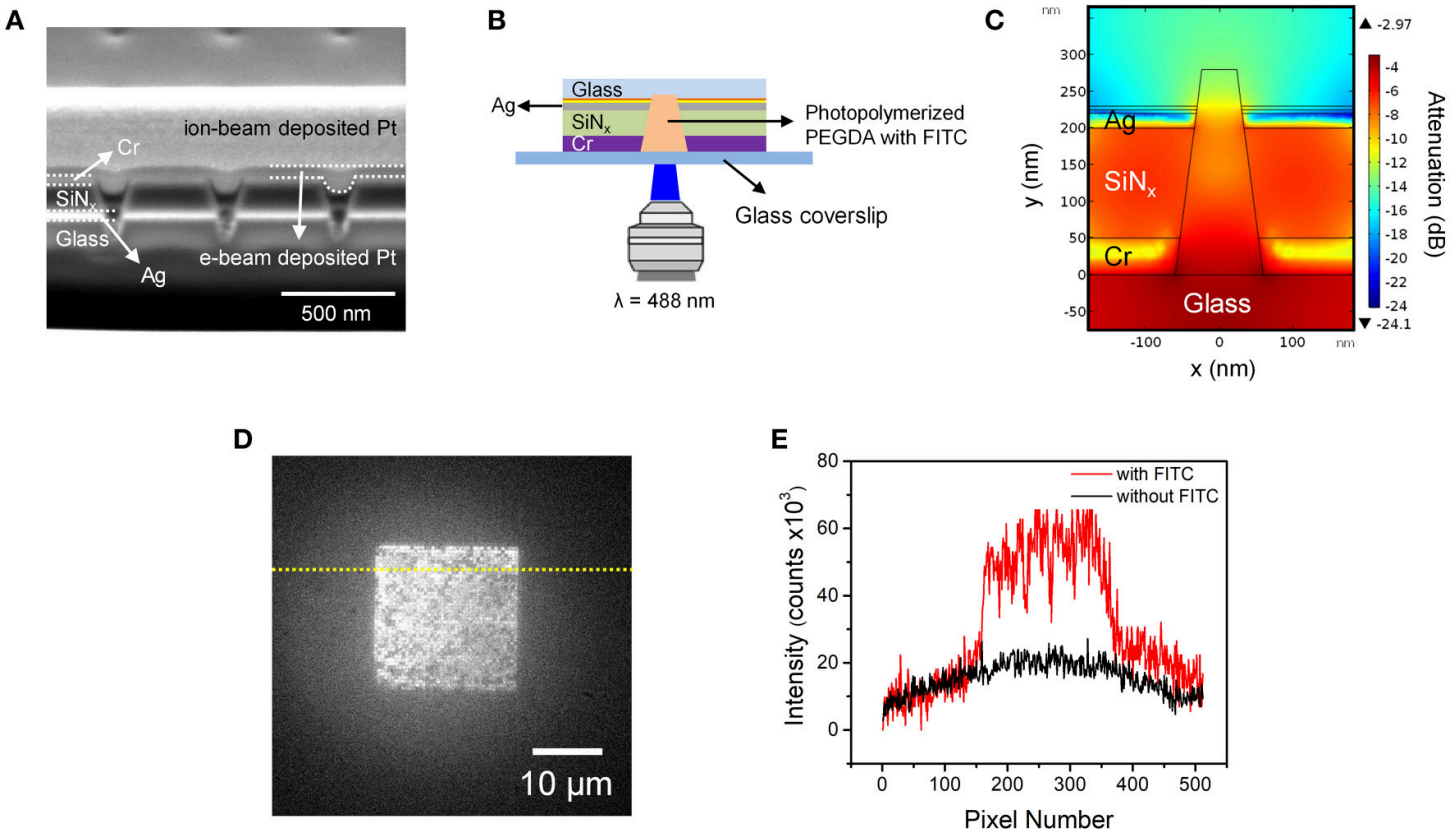
**(A)** Cross-sectional SEM image of photopolymerized PEGDA-FITC in nanopores. Positions and identities of functional layers are indicated on the left, while positions of e-beam and ion-beam deposited Pt structural layers, used to obtain cross-sectional images, are indicated on the right. **(B)** Schematic diagram of the fluorescence epi-illumination geometry. In this geometry the ZMW region is distal from the illumination plane. **(C)** Cross-sectional heat map of optical field amplitude in a conical nanopore obtained from a finite element simulation. **(D)** Fluorescence micrograph of a nanopore array containing photopolymerized FITC-PEGDA in the nanopores. **(E)** Fluorescence intensity profiles obtained along the dotted lines shown in **(D)** with (red) and without (black) FITC.

To further characterize the photopolymerization of PEGDA in ZMW nanopores, a fluorescent probe was added to the PEGDA monomer, and fluorescence was measured after photopolymerization. A 2.0 wt% solution of PEGDA monomer in ACN was prepared with 1 mM PI and 10 μM fluorescein isothiocyanate (FITC). The average number of fluorescent molecules occupying a single nanopore is given by 〈*n*〉_*pore*_ = *CVN*_A_, where *C* is the FITC concentration, *N*_A_ is Avogadro's number, and *V* is the volume of the conical frustum of a single nanopore. For the 2.2 aL volume of the conical frustum nanopores used in these experiments, single molecule occupancy, 〈*n*〉_*pore*_ = 1 is expected at 0.75 μM, meaning that under the conditions of this experiment, 〈*n*〉_*pore*_ ~ 13. Before photopolymerization, the ZMW nanopores were filled with the PEDGA solution and allowed to equilibrate at room temperature for 10 min, after which the PEGDA-filled nanopore array was exposed to UV radiation from the aligner for 30 min. Figure 3A shows a cross-section SEM image of adjacent nanopores containing FITC.

Importantly, in this experiment the array was irradiated from the large diameter side of the conical nanopores, in the fluorescence epi-illumination geometry illustrated in Figure 3B. To confirm that the FITC can be effectively excited once inside the nanopore, finite element simulations were conducted for this inverted configuration. Figure 3C shows a cross-sectional heat map of energy intensity obtained from a simulation of a single conical nanopore, confirming its ZMW behavior. Importantly, there is little attenuation of energy intensity inside the nanopore and nearly the entire volume of the nanopore can be excited. The simulation provided confidence that fluorescent radiation can be collected from the upper (Cr) surface of the ZMW array in order to characterize the fluorescence response of the FITC within the photopolymerized PEGDA-filled nanopores.

Figure 3D shows a low magnification, wide-area fluorescence micrograph of the ZMW array. Although there is some variation in intensity among the individual nanopores, the overall image indicates significant incorporation of FITC-impregnated photopolymerized PEGDA in the individual nanopores. Figure 3E shows a fluorescence intensity line profile obtained along the dotted line in Figure 3D, both with and without FITC. This result clearly shows isolation and photoactivation of the polyelectrolyte with fluorescent probes in each pore.

### Formation and Optimization of AgNPs Embedded in Polyelectrolyte Nanopore Array

Encouraged by the subwavelength control of the radial and axial field distributions provided by the ZMWs, we extended the fabrication of recessed Ag ring electrode array to include AgNPs and Ag^+^ embedded in the photopolymerized PEGDA. In principle, AgNPs arrayed in the dielectric PEGDA could form the basis of a metamaterial, and the presence of Ag^+^ would enable the electrochemical formation of nanofilaments connecting the AgNPs (Crouch et al., 2017; Chao et al., 2018). To understand the fabrication of nanopore-templated AgNPs embedded in PEGDA nanopillars, photopolymerization experiments were undertaken to investigate the influence of PEDGA concentration, Ag^+^ concentration, and the presence or absence of AgNPs on the formation of solid-polymer electrolytes within the nanopore templates.

**Figure 4:**
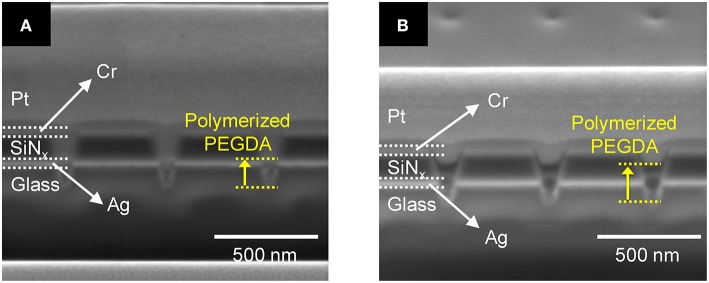
Cross-sectional SEM images of **(A)** 1.0 wt% and **(B)** 3.0 wt% photopolymerized PEGDA with 1 mM AgNO_3_ in a recessed Ag ring electrode array without AgNPs. The extent of the PEGDA photopolymer, after photopolymerization, is shown by the yellow dashed lines.

First, we tested the effect of PEGDA concentration in the presence of 1 mM AgNO_3_ but without AgNPs under optimized conditions. Figures 4A,B show cross-sectional SEM images of a recessed Ag ring electrode array obtained after photoirradiation of 1.0 wt% and 3.0 wt% PEGDA with 1 mM AgNO_3_, respectively. These concentrations were chosen to bracket the optimal conditions for PEGDA filling determined in initial tests, in which concentrations over 2.0 wt% PEGDA with 1 mM Ag^+^ were found to yield the best filling behavior in the absence of AgNPs. Photopolymerized PEGDA was observed to fill the nanopore only to the Ag ring for the 1.0 wt% monomer solution, but 3.0% PEGDA filled the nanopore up through the middle of the SiN_x_ layer, as shown in Figure 4B.

**Figure 5:**
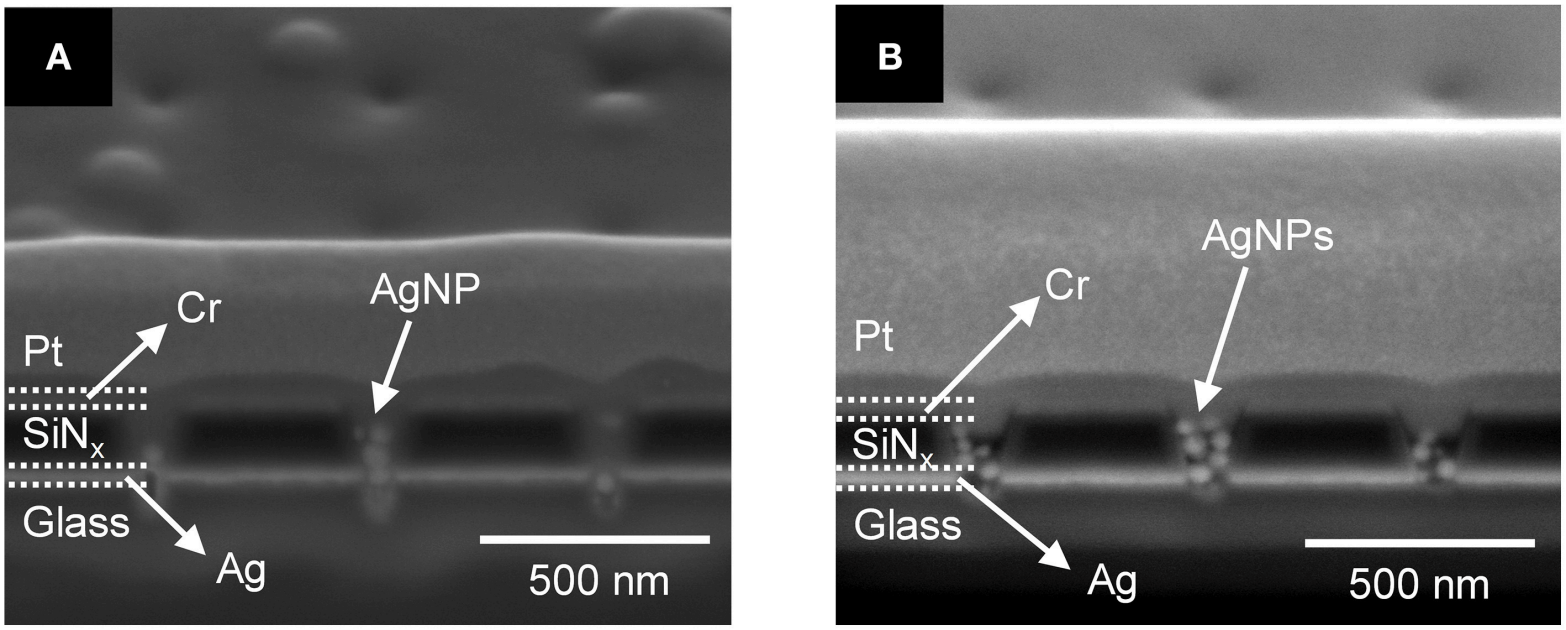
Cross-section SEM images of 1.0 wt% photopolymerized PEGDA in a recessed Ag ring electrode array with embedded AgNPs containing **(A)** 2 mM and **(B)** 1 mM AgNO_3_ salt.

Silver ions in the PEGDA are required for subsequent direct-write nanofilament formation and dissolution between AgNPs (Crouch et al., 2017; Chao et al., 2018), so we also investigated how Ag^+^ concentration affects the photopolymerization of PEGDA in the presence of AgNPs. Figures 5A,B show cross-sections of photopolymerized 1.0 wt% PEGDA with 2 mM AgNO_3_ and 1 mM AgNO_3_, respectively. Here, we included AgNPs in the PEGDA solution as opposed to the results in Figure 4 with no AgNPs. At 2.0 mM, Figure 5A, some AgNPs can be observed in the nanopore, but there is little to no polymerized PEGDA. However, at 1 mM Ag^+^ shown in Figure 5B, not only are individual stacked AgNPs observed, but they are in polymerized PEGDA. Surprisingly, comparison of Figures 5A,B shows that increasing the concentration of AgNO_3_ inhibits polymer filling of the nanopores. Given the nanoscale dimensions of the pores, an electrostatic screening effect is plausible, however understanding the mechanism giving rise to this effect will require further detailed studies in which polymer electrolyte characteristics and nanopore surface charge are carefully controlled. Nevertheless, this serves as experimental verification that in the presence of AgNPs, Ag-containing PEGDA monomer can be UV cross-linked to form AgNPs nanopillars embedded in the polyelectrolyte inside the nanopores.

**Figure 6:**
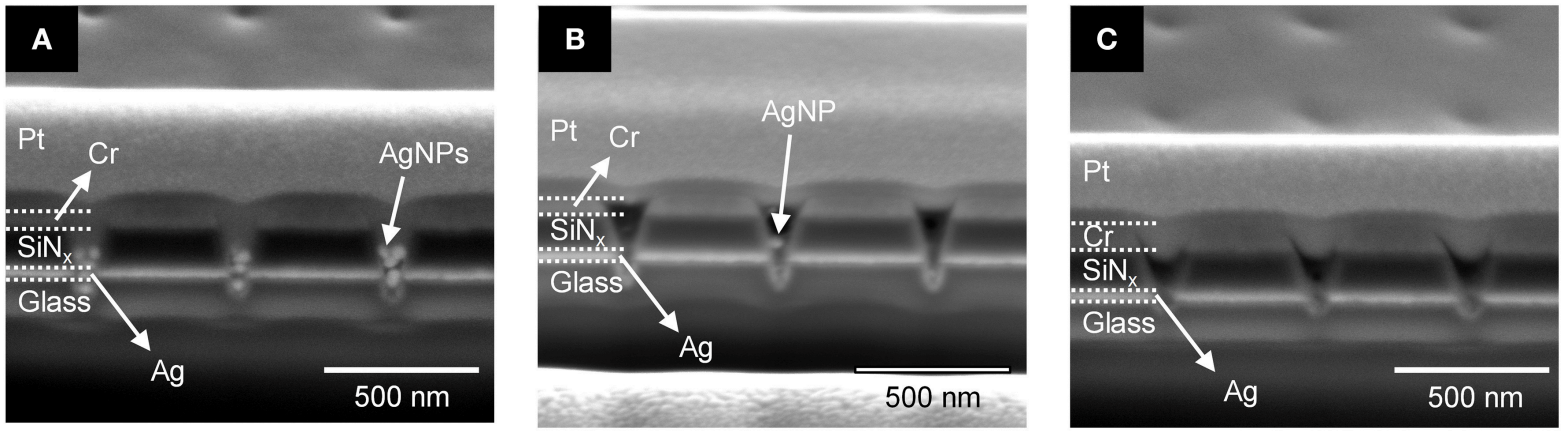
Cross-sectional SEM images of photopolymerized polyelectrolyte in a recessed Ag ring electrode array with embedded AgNPs for different PEGDA concentrations: **(A)** 0.5 wt%, **(B)** 1.0 wt%, and **(C)** 2.0 wt%.

Given the above result, in which lower Ag^+^ concentrations yield better fabrication of polymerized nanopillars, the photopolymerization behavior of PEGDA with AgNPs in a ZMW was also tested as a function of PEGDA concentration in the absence of Ag^+^ (i.e., no AgNO_3_). Figure 6 shows cross-section SEM images of PEGDA photopolymerized from solutions with various concentrations of monomer in the presence of AgNPs, but without Ag^+^. At the lowest concentration, 0.5 wt% PEGDA, Figure 6A, AgNPs fill the nanopore to the middle of the SiN_x_ insulator layer. Further increasing PEGDA concentration suppresses the number of AgNPs captured in the nanopore, *cf*. Figures 6B,C. One possible explanation is less physical crosslinking, which will decrease the likelihood of AgNP retention in the pore, although further experiments are needed to fully explore this interpretation. Interestingly, the PEGDA concentration dependence on pore filling is switched in the absence/presence of AgNPs. Higher PEGDA concentrations promote more extensive polymerization and therefore more pore filling in the absence of AgNPs, while lower PEGDA concentrations are more effective at pore filling when AgNPs are present. We tentatively assign this behavior to a volume-filling effect in the presence of AgNPs.

**Figure 7:**
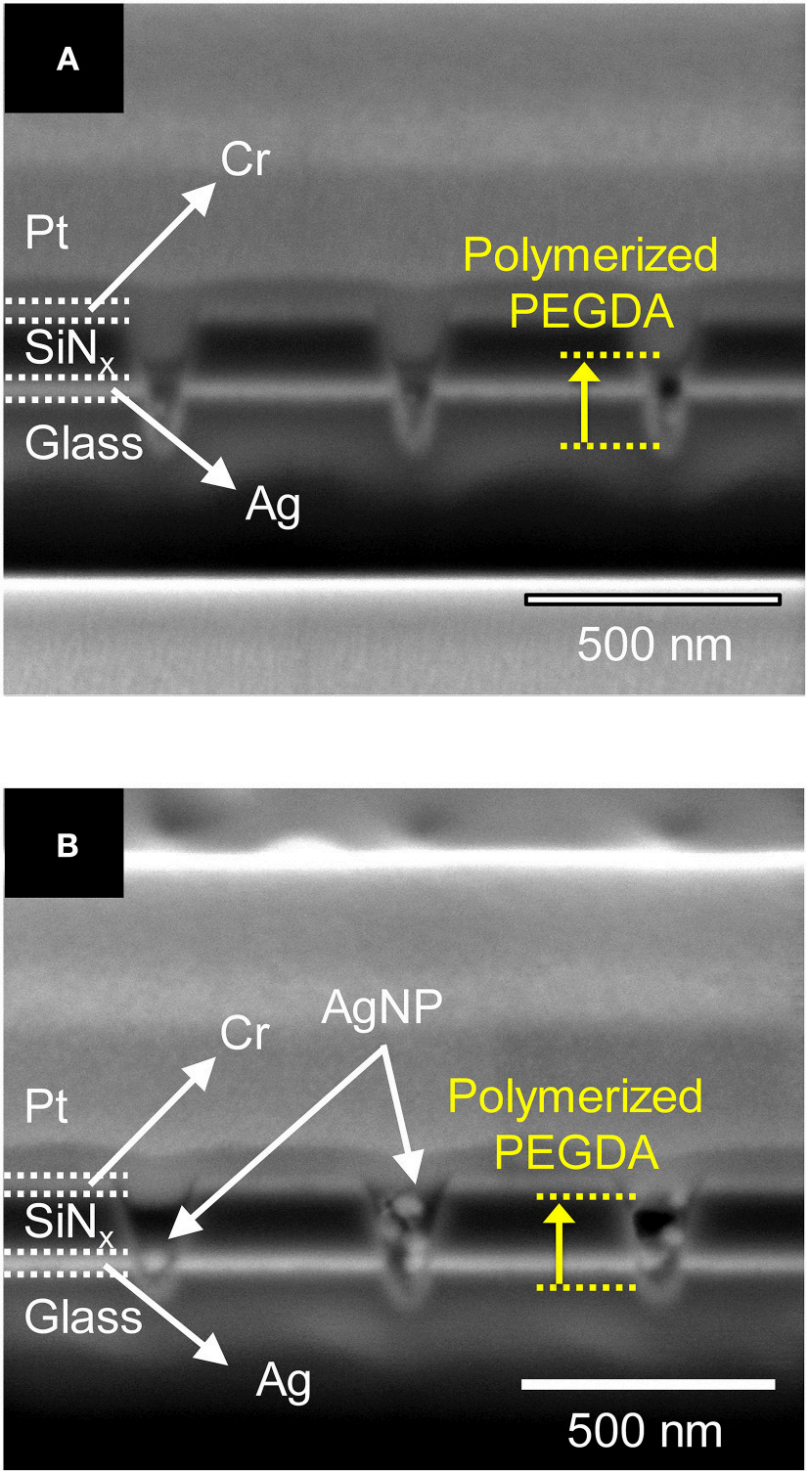
Cross-section SEM image of 2.0 wt% photopolymerized PEGDA and 1 mM Ag^+^ in a recessed Ag ring electrode array **(A)** without and **(B)** with embedded AgNPs.

Based on the above parametric experiments, we asked whether it might be possible to determine a single set of conditions that would yield satisfactory PEGDA polymerization behavior both in the absence and presence of AgNPs. Thus, we experimentally tested a preparation of 2.0 wt% PEGDA monomer with 1 mM AgNO_3_ salt in the presence and absence of AgNPs. The results shown in Figure 7 illustrate that this formulation yields photopolymerized polyelectrolyte nanopillars inside ZMW nanopores either without, Figure 7A, or with, Figure 7B, AgNPs. The resulting structures were effectively cross-linked by UV light, thus immobilizing the polyelectrolyte in the ZMW arrays. In the presence of AgNPs, this procedure clearly yields a nanopore-templated array of AgNP-containing solid-polymer electrolyte structures that could form the basis of a reconfigurable metamaterial.

## Conclusion

Templated pillars of AgNPs with nanometer-scale registration precision are of interest in nanophotonic and nanomanufacturing applications. Here, we describe a new fabrication strategy for producing ordered subwavelength arrays of AgNP nanopillars in PEGDA/Ag^+^ polyelectrolyte, using a ZMW to control and shape the spatial distribution of the confined electromagnetic field and, therefore, the volume occupied by photopolymerized PEGDA. Both chemical and ZMW geometric effects on the fabrication process were characterized. The influence of the optical cladding layer thickness on the ZMW-directed photopolymerization predicted through finite-element simulations was found to agree with experiment. We determined that careful control of the structure was necessary and sufficient to achieve well-controlled PEGDA photopolymer volumes. In addition, we characterized the solution conditions needed to produce well-templated PEGDA either with or without AgNPs and therefore identified optimal conditions for the preparation of nanopore-templated AgNP nanopillar assemblies in PEGDA/Ag^+^ polyelectrolyte. We believe that this approach employing *in situ* nanopore-templated fabrication of plasmonic and conductive nanostructures constitutes an exciting new platform for sequential formation/dissolution of nanofilaments through polyelectrolyte-confined nanopillar arrays of nanoparticles, thereby opening possible applications in actively reconfigurable metamaterials.

## Author Contributions

DG, SF-S, and PB designed the experiments. DH and GC performed the experiments. All authors contributed to analysis of the results, data analysis, and preparation of the final manuscript.

### Conflict of Interest Statement

The authors declare that the research was conducted in the absence of any commercial or financial relationships that could be construed as a potential conflict of interest.
